# A comprehensive description of self-reported health and life satisfaction in cardiac arrest survivors

**DOI:** 10.1186/s13049-021-00928-9

**Published:** 2021-08-21

**Authors:** Patrik Hellström, Kristofer Årestedt, Johan Israelsson

**Affiliations:** 1 Faculty of Health and Life Sciences, Linnaeus University, Kalmar, Sweden; 2 The Research Section, Region Kalmar County, Kalmar, Sweden; 3 Department of Internal Medicine, Division of Cardiology, Kalmar County Hospital, Region Kalmar County, Kalmar, Sweden

**Keywords:** Cardiac Arrest, Health, Heart arrest, Life satisfaction, Quality of life

## Abstract

**Background:**

Self-reported health and life satisfaction are considered important outcomes in people surviving cardiac arrest. However, most previous studies have reported limited aspects on health, often based on composite scores, and few studies have focused on life satisfaction. Investigating health aspects with a broad and detailed perspective is important to increase the knowledge of life after cardiac arrest from the perspective of survivors. In addition, the knowledge of potential differences in health among survivors related to place of arrest (in-hospital cardiac arrest; IHCA or out-of-hospital cardiac arrest; OHCA) is scarce. The aim was to describe and compare self-reported health and life satisfaction in IHCA and OHCA survivors.

**Methods:**

This observational cross-sectional study included adult cardiac arrest survivors six months after resuscitation, treated at five Swedish hospitals between 2013 and 2018. Participants received a study specific questionnaire including Health Index (HI), EQ-5D 5 Levels (EQ-5D-5L), Minimal Insomnia Sleeping Scale (MISS), Multidimensional Scale of Perceived Social Support (MSPSS), Hospital Anxiety and Depression Scale (HADS), and Satisfaction With Life Scale (SWLS). In order to present characteristics descriptive statistics were applied. The Mann-Whitney U test, chi-square test or Fishers’ exact test were used to compare differences in self-reported health and life satisfaction between in-hospital- and out-of-hospital cardiac arrest survivors

**Results:**

In total, 212 survivors participated. Based on scale scores and general measures, the median scores of health and life satisfaction among survivors were high: HI total = 29, EQ VAS = 80, and SWLS = 20. According to HI, most problems were reported for tiredness (37.3 %) and strength (26.4 %), while pain/discomfort (57.5 %) and anxiety/depression (42.5 %) where most common according to EQ-5D-5L. Except for EQ-5D-5L mobility (*p* = 0.023), MSPSS significant other (*p* = 0.036), and MSPSS family (*p* = 0.043), no health differences in relation to place of arrest were identified.

**Conclusions:**

Although general health and life satisfaction were good among cardiac arrest survivors, several prevalent health problems were reported regardless of place of arrest. To achieve an improved understanding of health in cardiac arrest survivors, it is important to assess specific symptoms as a complement to composite scores of general, physical, emotional, and social health.

## Background

Cardiac arrest (CA) is a significant health problem worldwide, associated with high risk of mortality [1]. During the last decades improvements in survival rates are reported [2], while less is known about the living conditions among the survivors, consequently resulting in poorly developed post-cardiac arrest care [3, 4].

According to the WHO, health is a multidimensional concept, defined as a state of complete, physical, psychological and social well-being, and not only absence of disease [5]. Health is considered an important outcome in people surviving CA [6]. Although suffering a sudden life-threatening event, survivors in general rate their overall health comparable to general populations [7, 8]. However, the variation is great and a significant proportion of survivors appear to suffer from serious health problems [2, 9]. Cognitive problems, fatigue and psychological distress, e.g. anxiety and depressive symptoms, are among the most frequently reported problems [10, 11]. Previous research indicates a difference between out-of-hospital (out-of-hospital cardiac arrest; OHCA) and in-hospital (in-hospital cardiac arrest; IHCA) CA survivors. Those surviving IHCA appear to report worse health and more psychological distress compared to OHCA survivors [8, 12, 13]. However, the clinical relevance of these findings remains unknown.

Most studies have reported limited aspects on health, often based on composite scores. A few studies have performed a more detailed analysis, identifying health problems especially in physical and psychological dimensions [7, 14]. However, since these studies have included selective groups of participants, e.g. OHCA survivors treated with targeted temperature management (TTM) [14] or with an implantable cardioverter defibrillator (ICD) [7], the results may not be transferable to the CA survivors in general. Additionally, aspects of social health and satisfaction of life are seldom considered.

Studies investigating health aspects with a broad and detailed perspective are important to increase the knowledge of life after CA from the perspective of survivors. Moreover, differences between OHCA and IHCA survivors need to be further investigated. Therefore, the aim was to describe and compare self-reported health and life satisfaction in IHCA and OHCA survivors.

## Methods

### Study design

This study had an observational and cross-sectional design. Data for the study was collected from the first patient follow-up after CA in a larger longitudinal study conducted between 2013 and 2018. The study conformed to the principles in the Declaration of Helsinki [15], and was approved by the regional ethical review board in Linköping, Sweden (No. 2013/235 − 31).

### Setting and procedure

The CA survivors were identified using the Swedish Register of Cardiopulmonary Resuscitation (SRCR), a nationwide quality registry including all CAs in Sweden where chest compressions and/or defibrillation is performed. Enrolment of participants was conducted at two university hospitals (Linköping and Gothenburg) and three county hospitals (Kalmar, Norrköping, and Växjö). The survivors were invited to participate during a registry follow-up telephone interview, performed by a cardiology nurse or resuscitation coordinator, at three to six months after suffering CA. Survivors interested to participate received written information, a consent form, and a study specific questionnaire six months after resuscitation. Those not responding received a reminder after six to eight weeks.

Inclusion criteria were being 18 years or older, suffering and surviving a CA due to cardiopulmonary disease, and being able to understand study instructions. In total, 317 survivors were eligible for inclusion and invited to participate. Of those, 66 chose not to participate and therefore 251 questionnaires were sent out. Despite the reminder, 39 did not return the questionnaire. Consequently, 212 survivors were included (67 % response rate). No significant differences in age or sex between participants and non-participants were detected. In addition, there were no significant differences in age or sex between the participants compared to those included in a previously published Swedish national registry study [13].

### Measurements

Data on time and place of CA were obtained from the SRCR. The questionnaire included information about demographics, financial situation, comorbidity, post-CA treatment and validated measures of health (Health Index, EQ-5D 5 Levels, Hospital Anxiety and Depression Scale, Minimal Insomnia Sleeping Scale, and Multidimensional Scale of Perceived Social Support), and life satisfaction (Satisfaction With Life Scale).

### EQ-5D 5 Levels (EQ-5D-5L)

The EQ-5D-5L measures health status and includes a descriptive five-dimension system and the EQ visual analogue scale (EQ VAS). The descriptive system includes mobility, self-care, usual activities, pain/discomfort, and anxiety/depression [16]. The five dimensions are rated using a five-level scale ranging from “no problems” (1) to “extreme problems” (5). Additionally, the scores can be dichotomized with a score ≥ 2 indicating the presence of a problem, regardless of severity. The EQ VAS is an overall measure of self-reported health status, with a score range from “the worst health you can imagine” (0) to “the best health you can imagine” (100). The EQ-5D-5L has previously been used to assess health in CA survivors [12] and is recommended as an outcome measure in the Core Outcome Set for Cardiac Arrest (COSCA) [17].

### Health Index (HI)

The HI is a generic measure of general health. The index is based on nine items including energy, temper, fatigue, loneliness, sleep, dizziness, bowel function, pain, and mobility. The responses are rated on a four-point scale ranging from 1 to 4, reflecting “major problems” (1) to “no problems” (4). The responses can be summarized to a total score ranging from 9 to 36. A higher total score indicates a better perceived general health [18]. Additionally, there are two overall items covering general health and perceived health during the last week, with responses from “very poor” (1) to “very good” (4). In addition to the index score, dichotomized item scores, with a score ≤ 2 indicating the presence of a problem, regardless of severity, were used in the present study. The HI has been used and validated in different populations [19], but has not previously been applied in CA survivors. In the present study, the internal consistency was satisfactory according to Cronbach’s alpha (0.80).

### Hospital Anxiety and Depression Scale (HADS)

The HADS is a screening measure developed to detect psychological distress in non-psychiatric patients. It consists of two subscales, one for anxiety (HADS-A) and one for depression (HADS-D) [20]. Each subscale consists of seven items, where each is scored from 0 to 3. The responses are summarized to a subscale score ranging from 0 to 21. Higher score indicates higher symptom levels. The following cut-off scores are recommended: 0–7 = normal range, 8–10 = suggested presence of mood disorder, 11–21 = probable presence of mood disorder [21]. The HADS has been used to screen for psychological distress in CA survivors and is recommended in international CA guidelines [22]. In the present study, the internal consistency was satisfactory according to Cronbach’s alpha for both anxiety (0.89) and depression (0.87).

### Minimal Insomnia Symptom Scale (MISS)

The MISS is developed as a screening tool for identifying insomnia, consisting of three items. Each item is scored from “no” (0) to “very severe problems” (4), with a total score from 0 to 12. A higher score indicates more problems with insomnia. A cut-off score of ≥ 6 is suggested for adults [23]. The MISS has demonstrated good measurement properties [24], but has not been used to screen for insomnia among CA survivors. In the present study, the internal consistency was satisfactory according to Cronbach’s alpha (0.76).

### Multidimensional Scale of Perceived Social Support (MSPSS)

The MSPSS is developed to measure perceived social support. The instrument includes 12 items and covering three dimensions; Family, Friends and Significant other. Each item is scored from “very strongly disagree” (1) to “very strongly agree” (7). The ratings on the items in each dimension are summarized and divided by four, which gives a possible score between 1 and 7 [25]. The MSPSS has demonstrated acceptable measurement properties [26]. In the present study, the internal consistency was satisfactory in all dimensions with Cronbach’s alpha values between 0.91 and 0.95.

### Satisfaction With Life Scale (SWLS)

The SWLS is a generic measure of life satisfaction [27]. The scale consists of five items rated on a five-point Likert scale, ranging from “strongly disagree” (1) to “strongly agree” (5). The item scores are summarized to a total score ranging from 5 to 25, with higher scores indicating more life satisfaction. The SWLS is widely used and has demonstrated good measurement properties [28]. However, the SWLS has not been used in CA populations. In the present study, the internal consistency was satisfactory according to Cronbach’s alpha (0.91).

### Data analyses

In order to present characteristics of the participant and included study variables, descriptive statistics were applied. The Mann-Whitney U test, chi-square test or Fishers’ exact test were used to compare differences in self-reported health and life satisfaction between IHCA and OHCA survivors depending on level of data. Statistical significance was set at p < 0.05. All statistical analyses were conducted using Stata 15.1 (StataCorp LP, College Station, TX, USA).

## Results

### Characteristics of participants

In total 212 CA survivors were included, with a mean age of 66.6 years (SD = 11.9). A majority were male (*n* = 162, 76.4 %) and suffered an IHCA (*n* = 136, 64.2 %). Those suffering IHCA were significantly older compared to OHCA (Δmean = 5.5, *p* = 0.002). Nearly half of the participants reported themselves to have comorbidities (*n* = 89, 42.0 %). The majority was born in Sweden (*n* = 202, 95.3 %) and were cohabiting (*n* = 174, 82.1 %). The median number of children was 2 (q1-q3 = 2–3), but OHCA survivors reported having more children compared to those suffering IHCA (*p* = 0.005). The majority of the survivors were retired (*n* = 134, 63.2 %) and reported having a good or very good financial situation (*n* = 190, 90.0 %). Most of the participants had been treated with percutaneous coronary intervention (PCI) (*n* = 139, 66.0 %) and one third were treated with implantable cardioverter defibrillator (ICD) (*n* = 76, 35.9 %). Half of the OHCA survivors (50.0 %) were working in contrast to one fifth (19.1 %) of the IHCA survivors (*p* < 0.001). A total of 53 (25.0 %) survivors had university education. No significant differences in gender, prevalence of comorbidities, educational level or economy were detected in relation to place of arrest (Table 1).

**Table 1:** Characteristics of participants

Variables	All (*n* = 212)	IHCA (*n* = 136)	OHCA (*n* = 76)	*p*-value
Sex, n (%)				0.980^b^
Male	162 (76.4)	104 (76.5)	58 (76.3)	
Female	50 (23.6)	32 (23.5)	18 (23.7)	
Age, mean (SD) [min/max]	66.6 (11.9) [30–90]	68.5 (11.0) [43–90]	63.0 (12.6) [30–85]	0.002^a^
Country of birth, n (%)				1.000^c^
Sweden	202 (95.3)	129 (94.9)	73 (96.1)	
Other country	10 (4.7)	7 (5.1)	3 (3.9)	
Number of children, Mdn (IQR)	2 (1)	2 (1.5)	2 (1)	0.005^a^
Cohabitation, n (%)				0.327^b^
Living alone	38 (17.9)	27 (19.9)	11 (14.5)	
Cohabiting	174 (82.1)	109 (80.1)	65 (85.5)	
Occupation, n (%)				< 0.001^c^
Working	64 (30.2)	26 (19.1)	38 (50.0)	
Retired	134 (63.2)	101 (74.3)	33 (43.4)	
Other	14 (6.6)	9 (6.6)	5 (6.6)	
Education, n (%)				0.276^c^
Lower than secondary school	11 (5.2)	8 (5.9)	3 (4.0)	
secondary school	60 (28.3)	41 (30.2)	19 (25.0)	
upper secondary school	88 (41.5)	59 (43.4)	29 (38.2)	
University	53 (25.0)	28 (20.6)	25 (32.9)	
Economics, n (%) ^d^				0.767^a^
Very good	43 (20.3)	30 (22.1)	13 (17.1)	
Good	147 (69.3)	90 (66.2)	57 (75.0)	
Poor	20 (9.4)	14 (10.3)	6 (7.9)	
Very poor	1 (0.5)	1 (0.74)	0	
ICD, n (%)				0.411^b^
Yes	76 (35.9)	46 (33.8)	30 (39.5)	
No	136 (64.4)	90 (66.2)	46 (60.5)	
PCI, n (%)				0.119^b^
Yes	139 (65.6)	84 (61.8)	55 (72.4)	
No	73 (34.4)	52 (38.2)	21 (27.6)	
Co-morbidity, n (%)				0.399^b^
Yes	89 (42.0)	60 (44.1)	29 (38.2)	
No	123 (58.0)	76 (55.9)	47 (61.8)	
Medical treatment for emotional distress, n (%)				0.520^b^
No	182 (85.8)	115 (84.6)	67 (88.2)	
Yes	30 (14.2)	21 (15.4)	9 (11.8)	
Healthcare contacts after SCA, n (%)				0.194^a^
No	124 (58.5)	84 (61.8)	40 (52.6)	
Yes 1 to 2 times	61 (28.8)	37 (27.2)	24 (31.6)	
Yes 3 to 5 times	23 (10.8)	11 (8.1)	12 (15.8)	
Yes more than 5 times	4 (1.9)	4 (2.9)	0	

^a^ Mann-Whitney U test, ^b^ Chi-squared test, ^c^ Fishers’ exact test, ^d^ Based on n = 211

IHCA = In-Hospital Cardiac Arrest, OHCA = Out-of-Hospital Cardiac Arrest

### Self-reported health

**Fig. 1:**
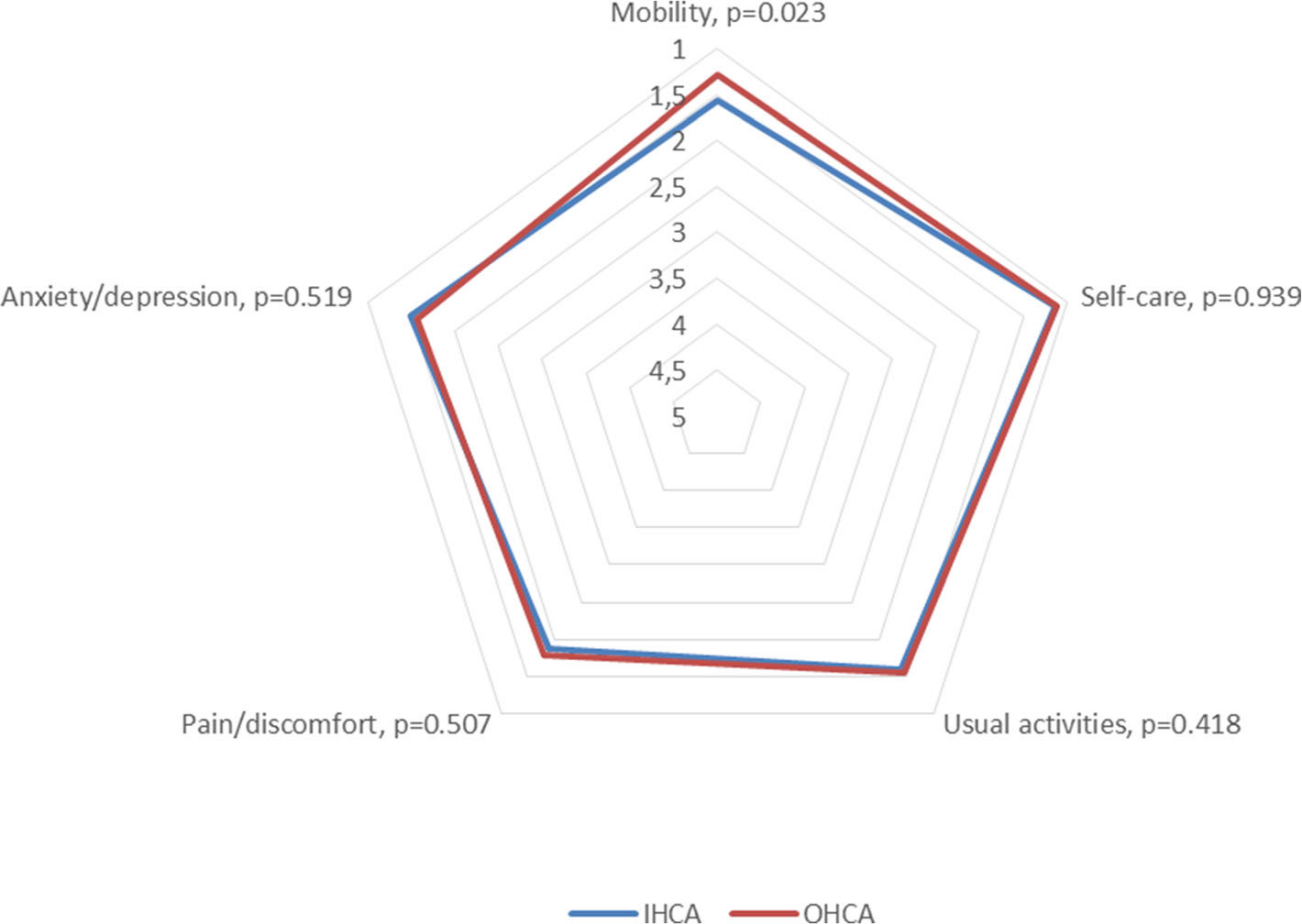
Severity of health problems in relation to place of arrest using the EQ-5D-5L. Mean values for IHCA and OCHA survivors (p-values based on Mann–Whitney U test). Higher values indicate more severe problems

The survivors reported overall high levels of general health status on EQ VAS (Mdn = 80, IQR = 15.5) (Table 2). Based on EQ-5D-5L descriptive system, a majority reported problems in the pain/discomfort dimension (*n* = 122, 57.5 %), while a minority reported problems in anxiety/depression (*n* = 90, 42.5 %), usual activities (*n* = 89, 42.0 %), mobility (*n* = 66, 31.1 %), and self-care (*n* = 20, 9.4 %). Extreme problems were reported in mobility (n = 2, 0.9 %) and usual activities (*n* = 1, 0.5 %) (Table 3). The IHCA survivors reported having more severe problems in mobility compared to those suffering OHCA (*p* = 0.023). No other differences in relation to place of arrest were detected using EQ-5D-5L (Fig. 1).

**Table 2:** Self-reported health and life satisfaction among participants

Variables	All (*n* = 212)	IHCA (*n* = 136)	OHCA (*n* = 76)	*p*-value^a^
Self-reported health				
EQ VAS, Mdn (IQR)	80 (15.5)	80 (20)	80 (12.5)	0.894
HI Total, Mdn (IQR)	29 (5)	29 (5)	29 (5.5)	0.663
HI Health last week, Mdn (IQR)	3 (1)	3 (1)	3 (1)	0.597
HI General health state, Mdn (IQR)	3 (0)	3 (0)	3 (0.5)	0.731
HADS Anxiety, Mdn (IQR)	3 (6)	3 (6)	2 (5)	0.902
Normal (0–7), n (%)	173 (82.0)	114 (83.8)	59 (78.7)	
Suggested presence of mood disorder (8–10), n (%)	25 (11.8)	13 (9.6)	12 (16)	
Probable presence of mood disorder (11–21), n (%)	13 (6.2)	9 (6.6)	4 (5.3)	
HADS Depression, Mdn (IQR)	1 (3)	1 (3)	1 (3)	0.933
Normal (0–7), n (%)	193 (91.4)	122 (89.7)	71 (94.7)	
Suggested presence of mood disorder (8–10), n (%)	11 (5,2)	10 (7.4)	1 (1.3)	
Probable presence of mood disorder (11–21), n (%)	7 (3.4)	4 (2.9)	3 (4.0)	
MISS, Mdn (IQR)	3 (1–5)	3 (2–5)	3 (1–5)	0.161
MISS < 6 (no problem), n (%)	170 (80.2)	108 (79.4)	62 (81.6)	
MISS ≥ 6 (problem), n (%)	42 (19.8)	28 (20.6)	14 (18.4)	
MSPSS significant other, Mdn (IQR)	6.75 (1.25)	6.5 (1.5)	7 (1)	0.036
MSPSS family, Mdn (IQR)	6.5 (1.5)	6.25 (1.75)	6.5 (1.25)	0.043
MSPSS friends, Mdn (IQR)	5.5 (1.25)	5.5 (2)	5.75 (1.5)	0.724
Life satisfaction				
SWLS, Mdn (IQR)	20 (4)	20 (3.5)	20 (4)	0.723

^a^ Mann-Whitney U test

*EQ VAS = EQ*  Visual Analogue Scale, *HADS* = Hospital Anxiety and Depression Scale, *HI* = Health Index, *IHCA* = In-Hospital Cardiac Arrest, *MISS* = Minimal Insomnia Symptom Scale, *MSPSS =* Multidimensional Scale of Perceived Social Support, *OHCA* = Out-of-Hospital Cardiac Arrest, *SWLS* = Satisfaction With Life Scale

**Table 3:** Prevalence and severity of health problems using the EQ-5D-5L

Variables	Extreme problems n (%)	Major problemsn (%)	Moderate problemsn (%)	Minor problemsn (%)	No problemsn (%)
EQ mobility	2 (0.9)	4 (1.9)	20 (9.4)	40 (18.9)	146 (68.9)
EQ self-care	-	1 (0.5)	4 (1.9)	15 (7.1)	192 (90.6)
EQ usual activities	1 (0.5)	6 (2.8)	17 (8.0)	65 (30.7)	123 (58.0)
EQ pain/discomfort	-	6 (2.8)	45 (21.2)	71 (33.5)	90 (42.5)
EQ anxiety/depression	-	5 (2.4)	10 (4.7)	75 (35.4)	122 (57.6)

*EQ-5D-5L* = EQ-5D 5 Levels

**Fig. 2:**
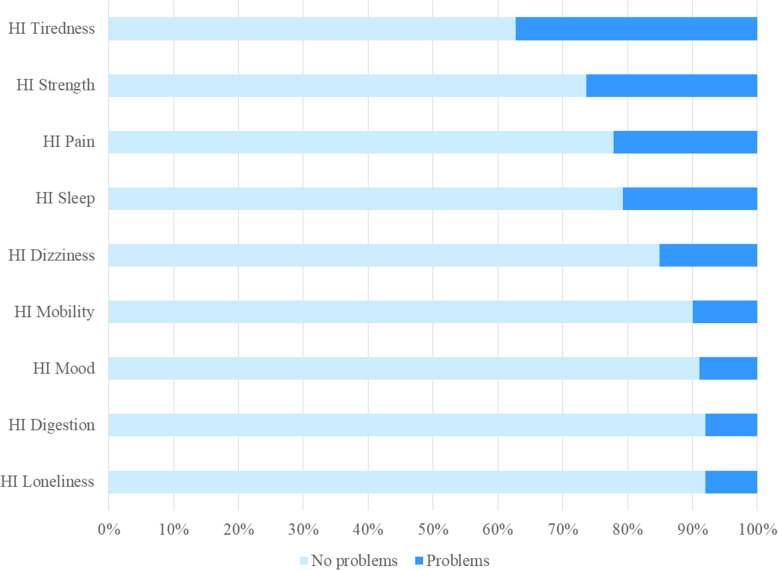
Prevalence of health problems among participants using the Health Index

**Fig. 3:**
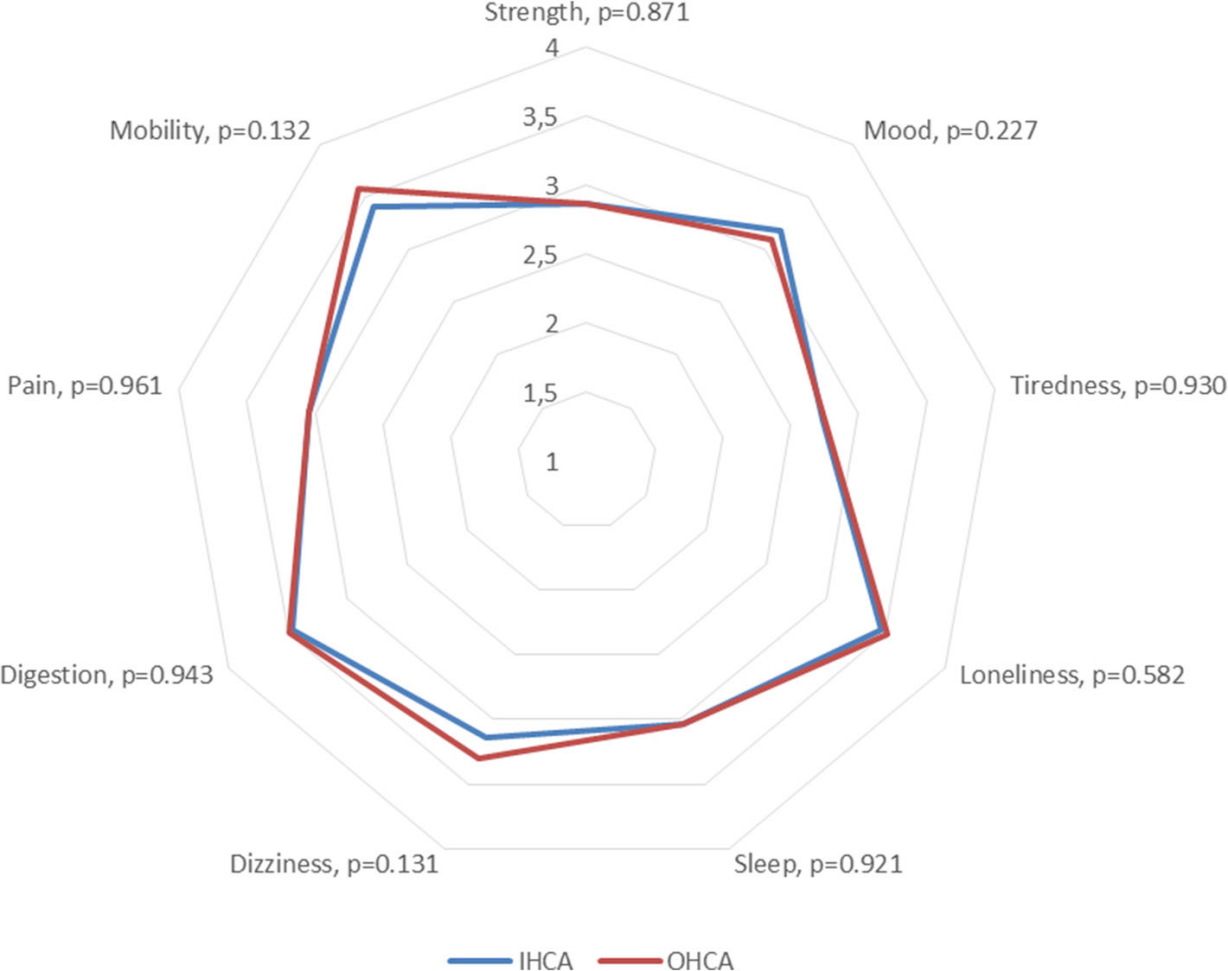
Severity of health problems in relation to place of arrest using the Health Index. Mean values for IHCA and OCHA survivors (p-values based on Mann–Whitney U test). Lower values indicate more severe problems

The median value of HI total score was 29 (IQR = 5). The corresponding median values for HI general health state and HI health last week were 3 (IQR = 0) and 3 (IQR = 1) respectively (Table 2). Most problems were reported for tiredness (*n* = 79, 37.3 %), followed by strength (*n* = 56, 26.4 %), pain (*n* = 47, 22.2 %), sleep (*n* = 44, 20.8 %), dizziness (n = 32, 15.1 %), mobility (*n* = 21, 9.9 %), mood (*n* = 19, 9.0 %), digestion (*n* = 17, 8.0 %), and loneliness (n = 17, 8.0 %) (Fig. 2). No differences were detected in relation of place of arrest using HI (Table 2; Fig. 3).

The median scores were 3 (IQR = 6) for HADS Anxiety and 1 (IQR = 3) for HADS Depression. Using the cut-off scores, 38 (18.0 %) vs. 18 (8.6 %) reported suggested/probable presence of mood disorder for anxiety and depression respectively. There were no significant differences for psychological distress in relation to place of arrest (Table 2).

Insomnia symptoms were reported by 19.8 % of the survivors. No differences in relation to place of arrest were detected using MISS (Table 2).

The CA survivors reported highest levels of social support in the dimension Significant other (Mdn = 6.75, IQR = 1.25), followed by Family (Mdn = 6.5, IQR = 1.5), and Friends (Mdn = 5.5, IQR = 1.75). The OHCA survivors reported significant higher levels of support in the dimensions Significant other (*p* = 0.036) and Family (*p* = 0.043) compared to IHCA survivors (Table 2).

### Life satisfaction

Using the SWLS the median score of life satisfaction in survivors was 20 (IQR = 4). No significant differences in relation to place of arrest were detected (Table 2).

## Discussion

This is one of the first studies presenting a comprehensive description of self-reported health including both IHCA and OHCA survivors. A significant proportion of the participants reported having health problems, mostly regarding tiredness, strength, pain and sleep. Despite their health problems, life satisfaction was rated as high. Only minor differences in self-reported health were detected related to place of arrest, where IHCA survivors reported more health problems with regard to physical and social health.

Self-reported health problems were not always detected using the scale scores and general health measures. Based on these measures (i.e., HI scale score, EQ VAS and HADS), the average health among survivors was high and similar to the general population. General measures of health are important since they allow easy comparisons to other populations. Moreover, such measures are reported to be associated with both morbidity and mortality [29, 30]. In contrast, a more detailed analysis of item-level data displayed a broad variety of health problems. To achieve an improved understanding of health among survivors it is important to assess specific symptoms as a complement to scale scores and general health measures.

One of the most prevalent and severe health problems in the present study was pain. This finding is in contrast to a previous Swedish study, investigating health problems in ICD-implanted CA survivors [7]. In that study, survivors reported less problems with pain even compared to a general population matched for sex and age. Experiences of severe pain are previously reported in qualitative CA studies [31, 32] and might be related to skeletal injuries caused by chest compressions. Pain is important to address during follow-up care, since it might cause immobility.

Sleep problems was reported by one fifth of the participants according to HI, which also is confirmed by results using MISS, where approximately one fifth scored 6 or higher (i.e., having insomnia symptoms). To our best knowledge, no previous study has specifically investigated sleep problems in CA survivors. It is well-known that sleep problems are common in heart disease. For example, in a study by Da Costa et al., as many as 36 % experienced clinical insomnia 5 weeks post-myocardial infarction [33]. Thus, the prevalence of sleep problems was lower in the present study. This could be explained by myocardial infarction being merely one of several different etiologies of CA, and that the higher proportion in Da Costa´s study could be due to increased anxiety close after the incident. The participants in our study may have had previous sleep problems, e.g. those suffering CA due to myocardial infarction. Another possible reason for the relatively high prevalence of sleep problems could be existential issues caused by suffering CA. It is important to continue to investigate sleep problems in CA survivors.

Less than 10 % of survivors reported loneliness regardless of severity using the HI. They also reported high levels of social support in all dimensions on the MSPSS. Similar levels of high social support were found by Alleman et al., in a study including patients who received an ICD due to heart failure [34]. Both the present study and their study showed that highest support was given by the *Significant other* followed by *Family* and *Friends*. It is possible that suffering a CA could trigger more social support.

EQ-5D-5L detected more problems with psychological distress than HI and HADS cut-off scores. However, the majority of the problems were considered minor. When studying health problems it is important to consider both prevalence and severity. Also, sensitivity and specificity of the applied measures need to be further investigated. For example, HADS might underestimate psychological problems, especially when a cut-off score is used. However, another possibility is that single item measures of psychological distress, e.g. EQ-5D-5L, might overestimate the prevalence of such problems.

The level of life satisfaction among participants were slightly lower compared to general populations [28, 35], but similar or higher compared to people suffering other diseases [36, 37], indicating that life satisfaction in general is good among CA survivors. In qualitative studies, experiences of an altered view of life are reported [32, 38]. People surviving CA might feel gratefulness for a second chance at life [31, 32]. Such feelings might compensate for potential health problems and might partly explain the high levels of general self-reported health and life satisfaction among CA survivors. Another possible explanation could be that Swedes in general reports high levels of life satisfaction compared to most other countries [39]. Since life satisfaction in general differs between countries and continents, it is likely that life satisfaction among CA survivors is dependent on where they live.

Few differences in self-reported health and none in life satisfaction were detected according to place of arrest. These results are in contrast to a previous Swedish study by Djarv et al. [13], reporting significantly lower self-reported health in IHCA survivors. However, although reporting significant differences, the effect sizes in their study were small.

### Limitations

This study has several limitations. Survivors with the poorest outcome, e.g. severe cognitive disability, were not included. This is a common reported limitation in CA studies [8, 12], since survivors with impaired cognition often have problems to complete self-reported measures. Selection bias regarding country of birth cannot be excluded since the vast majority were of Swedish origin. The results may not therefore be generalisable to those with poorest outcome and foreign-born. The outcome measures applied in this study are generic and have not been validated in CA survivors. Therefore, it is uncertain whether the reported health problems are a consequence of suffering CA or if the scales measure what is important for survivors. However, the EQ-5D-5L and the HADS are recommended outcomes in international guidelines for CA [6, 22]. There is a need for validation studies and developing condition-specific outcome measures for CA. Moreover, we lack information regarding the total number of survivors treated at the participating hospitals, including those not surviving until six months follow-up.

## Conclusions

Although severe health problems were uncommon and overall health and life satisfaction were good, several prevalent health problems were reported on an item level. Pain was one of the most common problems, which needs to be further investigated. Few differences related to place of arrest were identified. To achieve an improved understanding of health and life satisfaction among survivors, and to better identify health problems during post-CA care, it is important to assess specific symptoms as a complement to composite scores of general, physical, emotional, and social health.

## Data Availability

The data supporting the findings of this study are available from the corresponding author upon reasonable request.

## Abbreviations

CA: Cardiac arrest
COSCA: Core outcome set for cardiac arrest
EQ VAS: EuroQol visual analogue scale
EQ-5D-5L: EQ-5D 5 Levels
HADS: Hospital anxiety and depression scale
HI: Health index
IHCA: In-hospital cardiac arrest
ICD: Implantable cardioverter defibrillator
MISS: Minimal insomnia sleeping scale
MSPSS: Multidimensional scale of perceived social support
OHCA: Out-of-hospital cardiac arrest
PCI: Percutaneous coronary intervention
SRCR: Swedish register of cardiopulmonary resuscitation
SWLS: Satisfaction with life scale
TTM: Targeted temperature management
